# Elucidating the Influence of Chromosomal Architecture on Transcriptional Regulation in Prokaryotes – Observing Strong Local Effects of Nucleoid Structure on Gene Regulation

**DOI:** 10.3389/fmicb.2020.02002

**Published:** 2020-09-01

**Authors:** Thøger Jensen Krogh, Andre Franke, Jakob Møller-Jensen, Christoph Kaleta

**Affiliations:** ^1^Department of Biochemistry and Molecular Biology, University of Southern Denmark, Odense, Denmark; ^2^Institute of Clinical Molecular Biology (IKMB), Christian-Albrechts-University Kiel, Kiel, Germany; ^3^Institute of Experimental Medicine, Christian-Albrechts-University Kiel, Kiel, Germany

**Keywords:** bacterial nucleoid, gene co-expression, chromosomal architecture, transcriptional spilling, predicting supercoils

## Abstract

Both intrinsic and extrinsic mechanisms regulating bacterial expression have been elucidated and described, however, such studies have mainly focused on local effects on the two-dimensional structure of the prokaryote genome while long-range as well as spatial interactions influencing gene expression are still only poorly understood. In this paper, we investigate the association between co-expression and distance between genes, using RNA-seq data at multiple growth phases in order to illuminate whether such conserved patterns are an indication of a gene regulatory mechanism relevant for prokaryotic cell proliferation, adaption, and evolution. We observe recurrent sinusoidal patterns in correlation of pairwise expression as function of genomic distance and rule out that these are caused by transcription-induced supercoiling gradients, gene clustering in operons, or association with regulatory transcription factors (TFs). By comparing spatial proximity for pairs of genomic bins with their correlation of pairwise expression, we further observe a high co-expression proportional with the spatial proximity. Based on these observations, we propose that the observed patterns are related to nucleoid structure as a product of transcriptional spilling, where genes actively influence transcription of spatially proximal genes through increases within shared local pools of RNA polymerases (RNAP), and actively spilling transcription onto neighboring genes.

## Introduction

Coordinating the expression of functionally related genes in relation to environmental cues is pivotal for successful competition between species adapting to changing growth conditions (McAdams et al., 2004; Dillon and Dorman, 2010; Binder et al., 2016). This is evident through mutational experiments, and the optimization of gene expression is considered a fundamental driving force in evolution (McAdams et al., 2004; Seward and Kelly, 2018). Several mechanisms for optimization of co-expression are employed by prokaryotes to align and time expression of genes and to reduce energy spent on regulation, including co-regulation through association of transcription factors (TFs) and sigma factors (SFs; McAdams et al., 2004; Djordjevic, 2013). Genes related within highly defined functional groups are furthermore often organized into co-transcribed and co-regulated groups termed operons. In addition, operons and genes within less defined functional groups are further organized into larger domains (~20 kbp) of coordinated expression termed supra-operons (Junier et al., 2016, 2018).

The bacterial nucleoid requires extensive but reversible compaction to fit inside the confinements of the cell (up to >1.000 fold in length), while retaining accessibility of the entire chromosome (Krogh et al., 2018). Forces compacting the nucleoid include the association of DNA with specific proteins, transcription-induced DNA supercoiling, macromolecular crowding and entropy-driven depletion attraction, whereas the primary hypothesized expanding force is the coupling of transcription, translation, and membrane-insertion of membrane associated proteins, collectively referred to as transertion (Woldringh et al., 1995; Zimmerman, 2002; Cabrera et al., 2009; Mondal et al., 2011; Bakshi et al., 2015). Nucleoid compaction requires tight control to avoid detrimental effects on the cell during growth and division (Dillon and Dorman, 2010). In *Escherichia coli* the nucleoid is condensed during rapid exponential growth, with RNA-polymerases (RNAP) mainly located at a few highly active spatial loci (Cabrera and Jin, 2003; Cabrera et al., 2009). During entry into stationary growth phase the nucleoid of *E. coli* decondenses to cover most of the cell, with RNAP located all over the nucleoid (Cabrera and Jin, 2003). Finally, during late stationary growth, the nucleoid is highly condensed into a crystal-like structure together with the nucleoid associated protein (NAP) Dps (Frenkiel-Krispin et al., 2004; Kim et al., 2004). Based on DNA-DNA interaction studies, the *E. coli* nucleoid has been found to be divisible into four structured and two non-structured macrodomains (Valens et al., 2004). The number of DNA loops resulting from nucleoid compaction has been observed to be highly variable, between 40 and 500, with varying sizes (Postow et al., 2004; Stein et al., 2005; Dillon and Dorman, 2010). In conclusion, prokaryotes appear generally to have highly structured yet dynamically regulated nucleoids with distinct and relatively stable macrodomain conformations during optimal growth. These conformations are mainly established by supercoiled structures and binding of NAPs (Berger et al., 2010; Dillon and Dorman, 2010).

NAPs are a highly diverse family of proteins that share DNA-binding as the only unifying feature. The family includes a number of highly conserved members such as the HU family of histone-like proteins (Anuchin et al., 2011), and some species-specific members, such as H-NS and MukBEF (Dillon and Dorman, 2010; Krogh et al., 2018). NAPs generally exert their function by bending, winding, lassoing, or bridging DNA, thereby bringing distant genomic positions into close spatial proximity (Dillon and Dorman, 2010; Krogh et al., 2018). Due to the saturation of bacterial chromosomes with coding sequence, NAP binding sites often occur within transcribed regions. NAP binding at or in open reading frames may impact gene-regulatory regions, and indeed, many NAPs are considered important TFs (Dillon and Dorman, 2010). The intercellular levels of the different NAPs are highly diverse, and vary between different growth phases, underlining their roles in organizing the genome (Azam and Ishihama, 1999; Talukder and Ishihama, 2015).

Studies in eukaryotes suggest that inter-chromosomal co-expression of genes may be facilitated in part by nuclear structure, and consistent correlation can be observed across expression datasets from highly diverse conditions (Kustatscher et al., 2017). The organization of the prokaryotic genome suggests that similar relationships between co-expression of genes and nucleoid structure may be highly relevant for bacterial gene regulation (Jeong et al., 2004; Xiao et al., 2011; Weng and Xiao, 2014).

In *E. coli*, evolutionarily conserved gene pairs have been found to be located at conserved distances across multiple strains, namely at periods of 117 and 33 kbps (Wright et al., 2007; Mathelier and Carbone, 2010). Past studies in *E. coli* have investigated co-expression as a function of distance between genes over large microarray dataset collections, observing patterns, and defining periods of co-expression in *E. coli* as short-range (~20 kbps distance), medium-range (100–125 genes), and long-range (600–800 genes; Jeong et al., 2004; Mathelier and Carbone, 2010; Junier and Rivoire, 2016). Such periodic patterns, albeit with several different frequencies, have also been observed in *Buchnera* spp. (Viñuelas et al., 2007). However, such microarray studies, investigating correlations in expression change and distance, might be subject to a technical bias due to the probe design of microarray chips (Balázsi et al., 2003; Kluger et al., 2003; Koren et al., 2007).

In this study, we created and utilized RNA-seq data from defined growth phases of *E. coli*, in order to investigate the association between co-expression of genes and their relative position on the chromosome. We find recurrent sinusoidal patterns with region dependent frequencies, in correlation of pairwise expression as function of genomic distance. We investigate potential sources of the observed periodic increases in co-expression and rule out transcription-induced supercoiling gradients and operons. Furthermore, we find no immediate connection to specific binding profiles of various TFs, SFs, or NAPs. We observe that the identified patterns match existing data on DNA-DNA interaction frequencies and propose a model to explain the observed patterns through nucleoid structure and transcriptional spilling and discuss these findings in an evolutionary perspective.

## Materials and Methods

### Bacterial Growth and RNA Harvest

In order to obtain transcriptional data, RNA samples of *E. coli* BW25113 grown in rich media were purified at OD_600_ 0.2, 0.5, 1.2, 2.0, and 5.0 in duplicates, corresponding to early exponential, mid exponential, early stationary, mid stationary, and late stationary phases.

In short, 5 ml Lysogenic broth (or Luria Bertani broth; LB for short) w/o antibiotics were inoculated with a single colony from an ON LB agar plate with *E. coli* BW25113 and grown at 37°C ON. For each individual sample; 1 L flask containing 200 ml LB (2 L flask with 400 ml for early exponential sample) preheated to 37°C was inoculated to an OD_600_ of 0.05 and grown in a preheated 37°C water-bath with ~160 rpm aeration. OD_600_ was controlled regularly to confirm that culture was in the growth phase of interest. At the wanted OD_600_ 100 ml culture (200 ml for OD_600_ ~0.2) was flash-frozen using liquid nitrogen, spun down at >15.000 RCF for 10 min in a −4°C precooled centrifuge. Supernatant was removed and pellets stored at −80°C for no more than 5 days, before RNA purification.

RNA purification was done using chloroform-phenol extraction, on all samples individually. In short, cell pellets were resuspended in phenol (pH ~4.5) and chloroform before heated to 80°C for 2 min under vigorous shaking. The aqueous phase was extracted and transferred into 10x volume of −20°C cold 96% ethanol and left at −20°C for 20 min for RNA precipitation. Solution was spun down at 15.000 RCF for 1 h at −4°C. Solution was removed and RNA-pellet dried and left at −80°C.

RNA-library preparation and sequencing were outsourced, following the TruSeq Stranded Total RNA standard protocol from Illumina, using a HiSeq4000. Raw data has been deposited at the national center for biotechnology information (NCBI) gene expression omnibus (GEO) database under the accession number GSE153815.

### Computing the Pearson Correlation Between Bins Expressional Profiles and Estimate Periodicities Within

RNA-samples were sequenced using a Hi-seq 4,000, as 2x75bp paired-end reads. Raw reads were mapped to *E. coli* BW25113 (NZ_CP037857.1) or *E. coli* MG1655 (NC_000913.3), respectively, using Bowtie2 with local mapping settings (--local). Sequence alignment map (SAM)-files were converted to bam, sorted, and indexed using Samtools (v.0.1.19).

**Figure 1:**
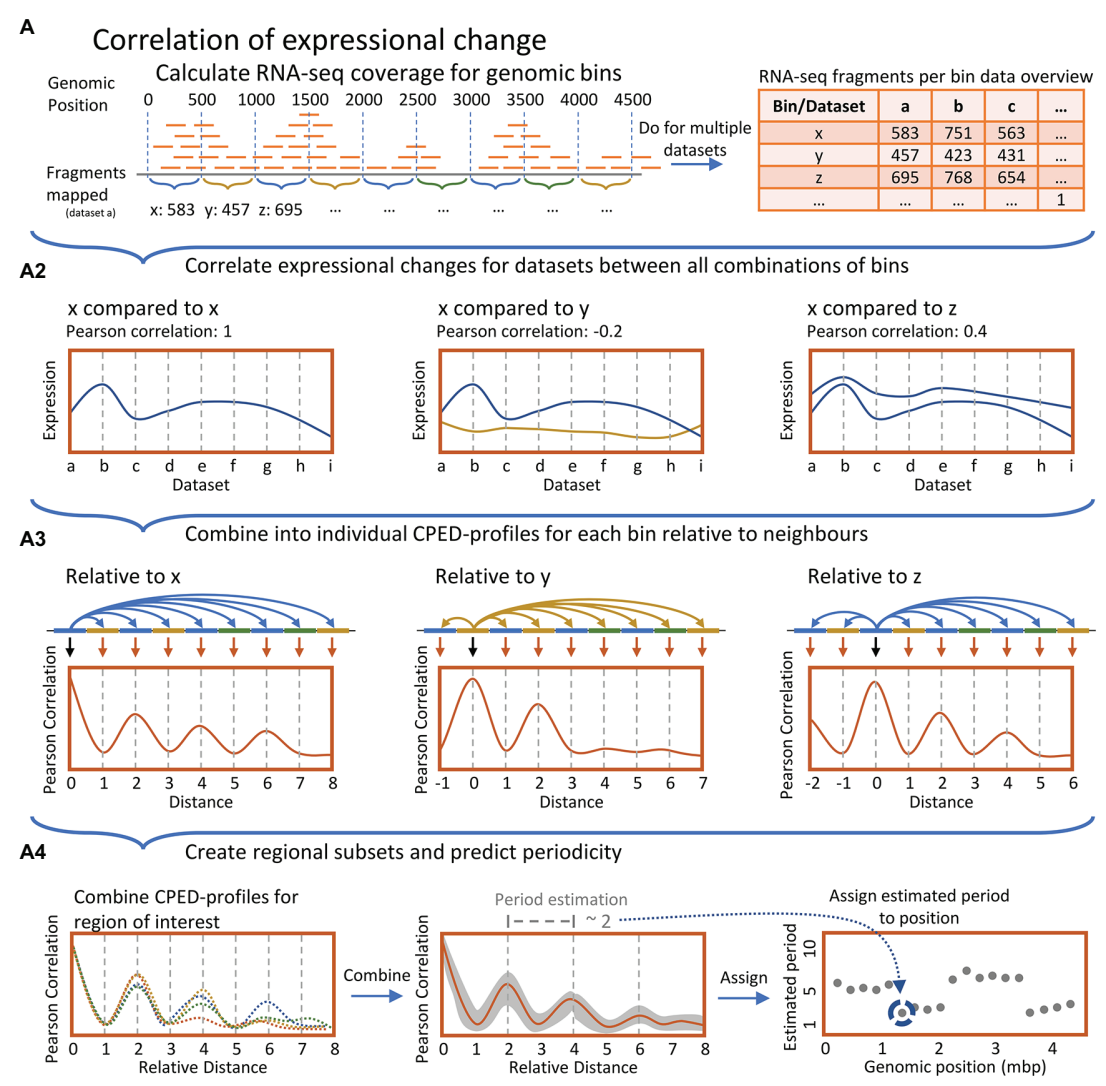
**(A)** Simplified data processing overview. Top panel: For all datasets, genomic coverage of RNA-seq data were calculated and summed for 1,000 bp genomic bins sliding at 500 bp. **(A2)**: Pearson correlation coefficients were computed relative to pairwise expression over all datasets between all bins. **(A3)**: Correlation coefficients for individual genomic bins relative to all neighbors were combined, yielding individual Expressional Correlation relative to Distance Profiles (CPED-Profiles) for all genomic bins, individually. **(A4)**: Multiple CPED-profiles were combined and averaged for bins within genomic regions of size 400 kbp. Subsequently, recurring periodicities were predicted through a Lomb-Scargle periodogram analysis, and the most statistically significant period for each region was mapped in a plot according to the center of the genomic 400 kbp region investigated.

To avoid gene length bias, 500 bp wide genomic features were created, spanning the entire genome. Genome coverage were calculated for all features, using the Rsubread (v.2.2.4; Liao et al., 2019), for each sample respectively, and normalized to fragments per million. Due to differing ribosomal RNA (rRNA)/transfer RNA (tRNA) depletions between the causing bias in the normalization, all bins associated with rRNA/tRNA/transfer-messenger RNA (tmRNA) expression were nulled (based on genome annotations: ASM584v2 and ASM435510v1). Furthermore, all correlation values related to any bin associated with rRNA/tRNA/tmRNA computed were substituted with an empty value. Biological duplicates averaged into single datasets for each growth phase. Finally, correlation of expressional change between bins were calculated for all possible combinations of bins using the R (v.3.6.3) built-in correlation function cor() with arguments use = “complete.obs” (Figure 1A).

To investigate periodicities of Pearson correlation between bins relative to distance, the average Pearson correlation for bins within windows of 400 kbp sliding at 5 kpb across the genome was calculated. Periodicities within these averaged correlation profiles were estimated using the Lomb-Scargle periodogram analysis R-package lomb (v.1.2; Ruf, 1999). Lomb-Scargle periodogram analysis was used due to its ability to detect rhythms in noisy incomplete data and the ability to ascertain the significance of estimated peaks using PNmax (Lomb, 1976; Scargle, 1982; VanderPlas, 2018). PNmax is defined as −log(*p*-value), hence the higher PNmax, the higher the probability of the estimated periodicity being valid. The distribution of PNmax values for all estimated periodicities across the genome observed within the transcriptional data acquired in this paper was compared to the PNmax distribution for estimated periods within 400 randomized datasets (Supplementary Figure S2). In short, data was randomized by assigning all genomic bins random unique genomic positions, followed by Lomb-Scargle periodogram analysis as described above.

### Transcriptional Factor, NAP Enrichment, and Sigma Factor Enrichment

Data from RegulonDB were used. Only TFs, NAPs, and SFs associated with more than a total of 20 genomic bins (+/− 500 bp of regulatory targets) were analyzed. Regulatory targets of TFs and SFs were obtained from RegulonDB (Release: 10.7 Date: 05/04/2020; Huerta et al., 1998; Santos-Zavaleta et al., 2019).

### Chromosomal Conformation Capture Comparison

For the investigation of any relation between DNA-DNA interaction frequencies and correlation of pairwise expression of genomic positions processed data from Lioy et al. (2018) were acquired from GEO database, accession GSE107301. Genomic bins used by Lioy et al. (2018) were 5 kbp wide, hence for this part correlation was calculated between bins of the same size, 5 kpb, created as described in 6.2.

### General

#### Software

All data were aligned to *E. coli* K-12 substrain BW25113 (Version NZ_CP037857.1), using Bowtie2 (v.2.3.5.1; with --local; Langmead and Salzberg, 2012) *via* whole genome sequences retrieved through the NCBI Nucleotide database. The output SAM files were converted to Binary Alignment Map (BAM) files and subsequently sort and indexed using Samtools (v.1.10; Li et al., 2009). Initial visual verification of data was done using SeqMonk (v.1.45.4). Fragments were mapped to 500 bp genomic bins using the R-package Rsubread (Liao et al., 2019). For data analyses, *R* was used. R-Packages: For general data tidying and wrangling the tidyr and reshape2 were used. Plots and data-visualizations were made using ggplot2 (Wickham, 2016). For general file and data manipulation Bash, Python, and Powershell was used. Explanatory figures made in power point.

#### Other Species

To investigate the presence of periodic patterns in other species, RNA-seq as raw fastq data from the NCBI GEO database was retrieved and analyzed as described in 6.2 top section, see Figure 1A. Data used from *Streptococcus sanguinis SK36* (Data from BioProject PRJNA381491 available at GEO database; El-Rami et al., 2018), and *Listeria monocytogenes* (Data from BioProject PRJNA270808 available at GEO database; Tang et al., 2015).

## Results

### Genomic Co-expression Correlates in Periodic Patterns

Transcription is highly regulated, with the main component of the transcriptional machinery being the RNAP (Saecker et al., 2011). The RNAP scans for promoters in a three-dimensional Brownian diffusion, where it stochastically binds and releases accessible DNA stretches until a matching promoter region is recognized and transcription is initialized (Wang et al., 2013). The bacterial nucleoid is a highly structured macromolecule that dynamically changes spatial organization in response to changing growth conditions (Valens et al., 2004; Cagliero et al., 2013). Interestingly, the distribution of RNAP across the nucleoid also changes dramatically during different growth conditions, suggesting a possible link between nucleoid structure and transcription regulation (Jin and Cabrera, 2006). The link is further underlined by the impact spatial proximity has on the efficiency of regulation *via* TFs (Pulkkinen and Metzler, 2013). An unbiased comparison of co-expression, of otherwise functionally unrelated genes, across different datasets obtained under different growth conditions, may highlight common co-expression patterns.

Previous studies investigating patterns in co-expression have been based primarily on micro-array data. However, as early studies have revealed a risk of microarray design bias in this type of study (Supplementary Figure S1; Jeong et al., 2004; Xiao et al., 2011; Junier and Rivoire, 2016), we created transcriptomic data based on RNA-seq (materials and methods). In addition, previous studies either investigated entire large databases of micro-array data or only a few seemingly arbitrarily chosen datasets, without taking growth-specific changes to the nucleoid into account, possibly missing important structural features due to noise (Jeong et al., 2004; Xiao et al., 2011; Junier and Rivoire, 2016). Lastly, some of the previous studies erroneously consider distance as ordinal, by gene order, rather than numeric, by genomic position, leading to a misleading interpretation of period in the context of genomic distance (Supplementary Figure S1). To determine the potential effect of nucleoid structure in co-expression patterns, we investigated data from few, but well-defined growth phases, with reported changes in nucleoid compaction state (i.e., between exponential and stationary growth). This was done in order to obtain maximum dataset variation in both changes in expression and nucleoid structure (Krogh et al., 2018). This way patterns in co-expression relative to genomic position can be related to changes in nucleoid structure when moving from highly to less condensed nucleoids.

In order to investigate distance determined co-expression patterns, the acquired transcriptomic data was divided into bins of 500 bp in size. Subsequently changes in expression, induced by a change in growth phase, were compared between all possible combinations of these genomic bins and the correlation coefficients of pairwise expression were related to the distance between bins (Figures 1A-top panel, A2). This yielded a Correlation of Pariwise Expression relative to Distance Profile (CPED-Profile) for all bins, respectively (Figure 1A3). Periodic patterns in the data were analyzed by combining CPED-profiles for all bins within a given genomic window followed by an estimation of the observed wavelength of the averaged profile. Wavelength estimation was conducted by use of Lomb-Scargle least-squares frequency periodogram analysis (Figure 1A4; Lomb-Scargle for short. See methods; Lomb, 1976; Scargle, 1982; Ruf, 1999; VanderPlas, 2018).

**Figure 2:**
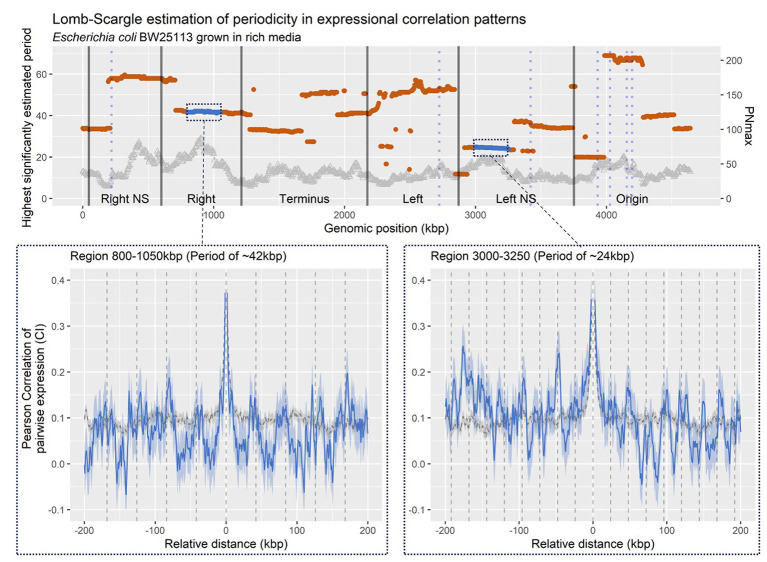
Lomp-Scargle Periodogram frequency estimation. **Top panel**: Estimated frequencies of correlation profiles at a relative distance of +/− 200 kbp, for bins within 400 kbp genomic regions, sliding at 5 kbp. Dots are positioned at the genomic region middle. Black vertical lines indicate structured and unstructured (mixed) macrodomains in *Escherichia coli* as defined by Valens et al. (Valens et al., 2004; corrected for strain BW25113 coordinates). Blue vertical lines indicate the position of ribosomal RNA (rRNA) operons in *E. coli* BW25113. Left y axis indicates PNmax [−log(*p*-value) of the estimated period], PNmax > 3, equal *p* < 0.05. **Bottom panel**: Averaged CPED-profiles of regions (blue line), or for entire genome (gray dashed), with confidence interval (CI; 95%) of true mean. **Left panel**: Region 800–1,050 kbp, within Right structured macrodomain, with a predicted period of ~42 kbp. **Right panel**: Region 3.000–3.250 kbp, within Left unstructured macrodomain, with a predicted period of ~24 kbp.

In brief, the Lomb-Scargle method compares modeled sine/cosine frequency functions within a defined window of possible frequencies, to the experimental data and estimates the goodness of fitness by computing the least-squares. Hence, each specific region has unique estimates for all frequencies within the arbitrarily chosen range of 10–70 kbps. In Figure 2, the frequency with the highest fitness is reported for each specific region. However, we note that there may be sub or super frequencies in each region with almost as high estimated fits, explaining the abrupt changes in highest estimated period. The wavelength estimations across the entire genome (Figure 2-top panel) shows multiple large regions with a conserved estimate of wavelength, somewhat matching those defined prior by Valens et al. (2004). The regions show statistically significant periodicities estimated through PNmax defined as −log(*p*-value), as compared to shuffled genomes (Supplementary Figure S2). For example, at position 0–200 kbp, which shows a periodic pattern with an estimated period of 33 kpb, and terminus showing a roughly 35 kbp wavelength, as proposed based on *matS* domains (Dupaigne et al., 2012). This is indicative of recurring increases in co-expression for genomic bins relative to distance between bins. When looking closer at specific regions, it is evident that periodicities in co-expression are present as wavelike patterns in correlation relative to distance (Figure 2-bottom panel).

The observed change in averaged CPED-profile depending on genomic position suggests a systematic organization of the genome. Two known structural mechanisms, which could explain such high correlation in expressional change between neighboring genes are; (1) the transcriptional clustering of genes in operons or (2) the impact of supercoiling gradients created by active transcription and/or replication. However, operons can readily be excluded as the source, since only 13 operons larger than 10 kbp exist in the *E. coli* genome with the largest at 17.840 bp (RegulonDB; Operons Release: 10.6 Date: 10/04/2019; Huerta et al., 1998; Santos-Zavaleta et al., 2019). This leaves mechanism (2) to be further explored.

### Patterns in Co-expression Are Independent of Transcription-Induced Supercoiling Gradients

**Figure 3:**
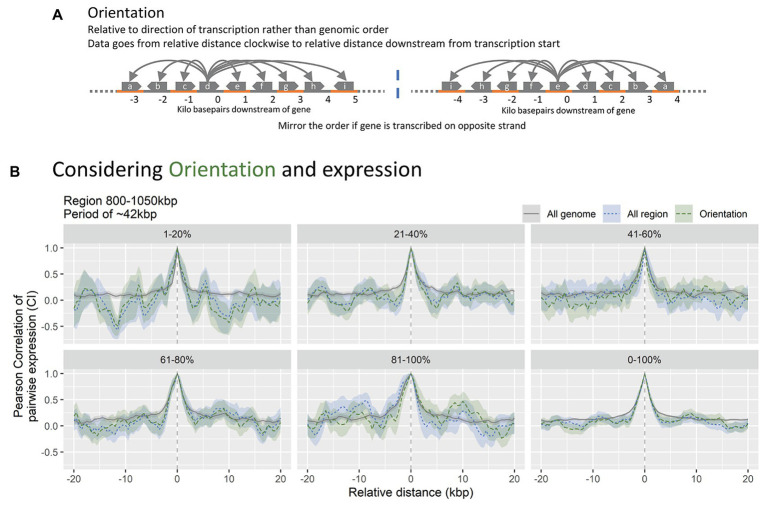
**(A)** Simplified data mutation overview. The distance variable of the CPED-profiles is converted to distance relative to transcription orientation of the bin, rather than being relative to the genomic position. Notice how the named bins change order upon mirroring, negating the original distance. **(B)** Data subsets relative to expression strength and orientation, for region 800–1,050 kbp, shown in Figure 2. Notice no significant changes to the CPED-profiles when accounting for orientation (blue vs. green).

Transcription-induced supercoiling gradients are considered a central regulatory mechanism since more than 2.000 genes in *E. coli* are sensitive to DNA supercoiling (Blot et al., 2006; Marr et al., 2008). Such gradients are generated by the accumulation of positive supercoiling in front of the transcribing RNAP, which promote expression of downstream genes, and negative supercoiling trailing the transcribing RNAP, which will inhibit transcription of upstream genes (Liu and Wang, 1987; Ma et al., 2013). Since transcription supercoiling gradients are dependent on the orientation of transcription, the distance to neighboring bins in the CPED-profiles must be analyzed relative to transcription orientation, rather than genomic position in order to investigate the potential contribution of supercoiling gradients. In order to do this, data bins were assigned an orientation according to the transcriptional direction of annotated gene(s) spanning and/or starting/ending within the bin. Bins without any assigned orientation or assigned with both clockwise and counter-clockwise orientations (relative to genomic coordinate) were discarded (~2/3rds of bins). Subsequently, all bins with a counter-clockwise orientation were mirrored to correctly indicate position relative to transcription (Figure 3A). Besides the orientation dependency, supercoiling is further related to the expressional strength. To further account for this, bins were divided into five percentiles of equal size according to their expressional strength (Figure 3B).

If the contribution of supercoiling gradients to the observed gene expression patterns is significant, the expected Pearson correlation of bins aligned to transcription orientation must be high downstream, relative to interval 0, whereas negative correlation is expected upstream. We do not observe an increase in co-expression upstream of direction of transcription-oriented bins compared to non-orientated data for the region or the entire genome. The lack of changes to co-expression relative to orientation of transcription suggests that transcription-induced supercoiling gradients are relieved instantly, possibly through DNA gyrase or Topoisomerase IV (Sharma and Mondragón, 1995; Schvartzman et al., 2013). This is in accordance with observations that supercoiling gradients lack during exponential phase where the nucleoid is condensed (Lal et al., 2016). The periodic pattern within the region 800–1,050 kbp is present when accounting for orientation, indicating that transcription-induced supercoiling is not the direct mechanism behind the observed patterns (Supplementary Figure S3). The exclusion of transcription-induced supercoiling is further supported by previous observations that the influence of supercoiling generated gradients decrease over distances of roughly 10 kbp, and that it has limited propagation outside domains of 10 kbp size as well (Postow et al., 2004; Sobetzko, 2016). When accounting for expression strength, the pattern amplitudes and frequencies however seem to vary, but due to the high amount of noise it is difficult to conclude any relation to expression strength (Supplementary Figure S3).

Having investigated structural mechanisms, which could explain the observed patterns in co-expression, we turn to another well-described mechanism behind co-expression, namely TFs modulating transcription of target-genes in bulk.

### Patterns in Co-expression Are Independent of Association With Transcription Modulating Proteins

Co-expressional patterns between distantly positioned genes on the genome have historically been attributed to TFs. These are proteins that modulate transcription through association to specific motifs and/or DNA structures (Djordjevic, 2013). TFs commonly modulate groups of genes associated with distinct environmental cues. An example is fumarate and nitrate reduction regulatory protein (FNR) that mediates transition from aerobic to anaerobic conditions by inducing genes related to anaerobic metabolism, and inhibits genes related to aerobic metabolism (Salmon et al., 2003; Kang et al., 2005; Myers et al., 2013). In this section, we also consider SFs, which are proteins that mediate selective promoter recognition by the RNAP and thereby modulate transcription as well (Saecker et al., 2011; Svetlov and Nudler, 2013; Duzdevich et al., 2014). In addition, the NAPs, which confer genome structuring, are considered gene-regulatory proteins since they often have a major impact on the transcription profile (Dillon and Dorman, 2010). Thus, in the following paragraph, the term TF includes both NAPs and SFs.

To investigate the relation between high co-expression and proximity known regulatory targets of the modulating proteins, we obtained information about TF regulates from RegulonDB (TF – gene interactions and Sigma – gene interactions Release: 10.7 Date: 05/04/2020; Huerta et al., 1998; Santos-Zavaleta et al., 2019).

**Figure 4:**
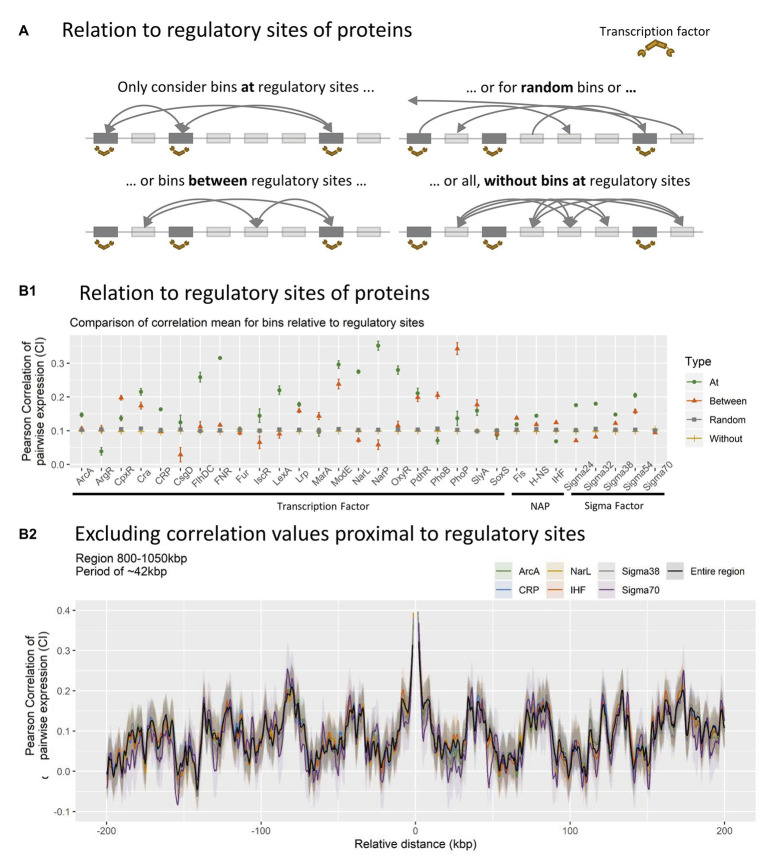
**(A)** Schematic overview of how different data subsets were created. All bins within 500 bp of nucleoid associated protein (NAP)/transcriptional factor (TF) regulatory site profiles (BS), were identified and the expressional correlation distributions was calculated for all BS-profiles individually (At). This was compared to mutated binding site profiles (between), random binding site profiles (random), and the overall distribution without the coefficients at binding sites (without). **(B)** Relation between protein binding and correlation coefficients. **(B1)**: Distributions of correlation coefficients within subsets. All bins associated with binding (At, green circle), All bins exactly between bins with associated binding (between, red triangle), average distribution of 100 random (with *N* = *N* ^At^ = *N* ^Between^) sets of bins (random, gray square), or total distribution between all bins NOT associated with binding (without, yellow line). **(B2)**: Impact of excluding bins at binding sites for TFs/sigma factors (SFs)/NAPs with more than 20 binding sites within the 800–1,050 kbp region.

The co-expression was compared between different bin subsets for each type of TF, respectively (see Figure 4 panel A for visual explanation). Co-expression of bins within 500 bp on either side of a regulatory target was considered as associated with binding sites (Termed *At*). These bins were compared to bins associated with mutated mock binding sites (Termed *Between*), created by moving real binding sites to positions between real sites in a clockwise manner. These were further compared to 100 randomly generated bin-subsets of equal size to the original binding site data (Termed *Random*), and mutated data where co-expression was computed for all bins without any association to the modulating protein in question (Termed *Without*).

The expected outcome of TF mediated co-expression would be a higher averaged correlation for bins at regulatory targets compared to all other subsets. This is indeed observed for TF’s ArcA, Cra, FlhDC, FNR, IscR LexA, Lrp, ModE, NarL, NarP, OxyR, H-NS, Sigma 24, Sigma 32, Sigma 38, and Sigma 54, suggesting that bins associated with binding of these specific TFs are indeed co-regulated during the investigated growth phases, as expected. However, this approach only identifies genome-wide co-expression of bins and does not sufficiently account for the observed periodic patterns in co-expression observed locally.

To investigate the impact of TFs at the local genome scale, bins associated with TF regulation were excluded from the averaged CPED-profile. This way any impact on co-expression conferred by the TF would be eliminated, and a diminished pattern would be expected in the case of TF binding impacting the observed periodic patterns. Six of the 32 TFs investigated were associated with more than 20 bins (of 500 possible) within the specific region shown in Figure 4B2 and were chosen for analysis. None of the six TFs show major impact on the observed periodic pattern after exclusion of TF-associated bins. The lack of major changes to the periodicity, indicates that TF association is not the primary mechanism behind the observed patterns. Hence the patterns in co-expression might emerge through a less known, but perhaps fundamental mechanism, which depends solely on the spatial structure of the nucleoid. The lack of major changes when excluding NAPs does not exclude the possibility of their involvement in the observations through genome structuring; it does simply indicate that their regulatory impact is not significant for the periodicities in transcription.

### Transcriptional Spilling, Expression Strength, and Correlation Pattern of Expression Change

**Figure 5:**
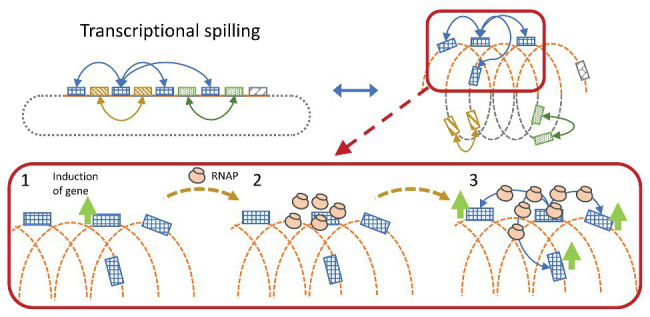
Simplified illustration of transcriptional spilling. Genes far from each other in the linear genome may be spatially close due to compaction of the genome. Gene induction leads to RNA polymerase (RNAP) recruitment, whereby the increase in local concentration of RNAP might spill onto spatially close genes increasing the chance of successful gene transcription initiation.

**Figure 6:**
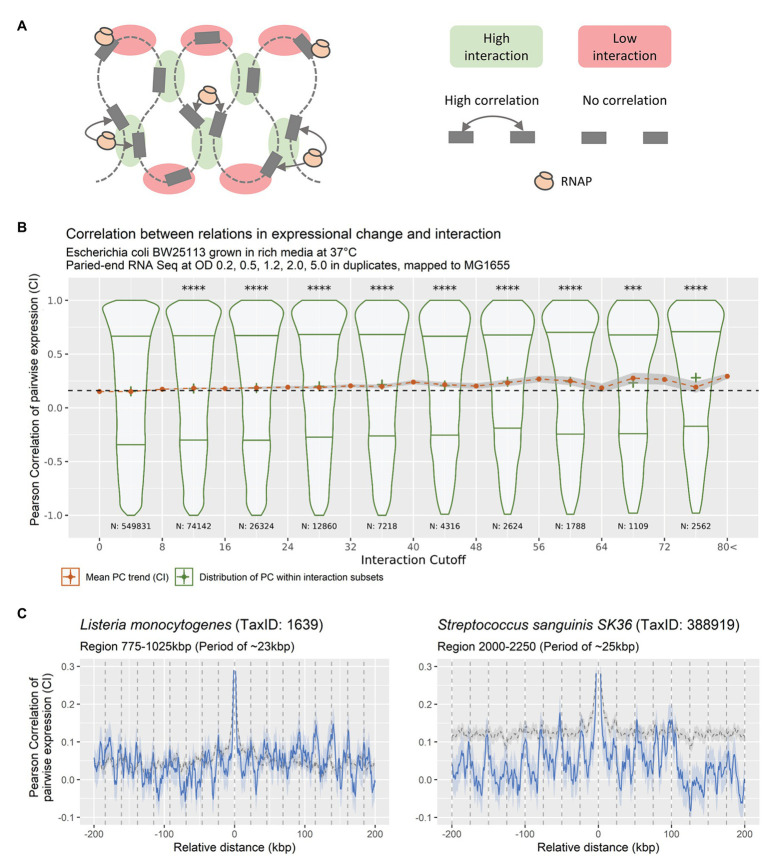
**(A)** Theoretical relation between DNA-DNA interaction frequencies and correlation of expression change between bins. If a local pool of RNAP is shared between spatially close genes, any changes to this pool will result in a coordinated response of the genes within the pool. **(B)** DNA-DNA Interaction relative to pairwise expression correlation (bin size 5 kbp), for DNA-DNA interactions of bins with a linear distance of more than 20 kbps. Violin-plots showing the distribution of expressional correlation for genomic positions grouped in eight distributions of inequal size according to increasing DNA-DNA interaction, with lower and upper quantiles and cross at mean (green distributions). Red dots at mean +/− confidence interval (95%) for expressional correlation grouped in 21 unequal-sized groups based on DNA-DNA. Stars indicate significance of distribution means being equal to the distribution with the lowest DNA-DNA interaction (^****^p < 0.00005 using a pair-wise Wilcoxon test, adjusting for multiple testing using Bonferroni, most were at *p* < 2e^−16^). **(C)** Averaged CPED-profiles showing mean and 95% confidence interval for true mean for data retrieved from NCBI GEO, mapped and analyzed locally. Gray dashed line indicates averaged CPED-profile for the entire genome. Blue solid line indicates averaged CPED-profile for the specific local regions as indicated in subtitle. Left panel: Data from BioProject PRJNA270808 (Tang et al., 2015), showing periodic patterns of ~23 kbp, within region 775–1,025 kbp of the Gram-positive *Listeria monocytogenes*. Right panel: Data from BioProject PRJNA381491 (El-Rami et al., 2018), showing periodic patterns of ~25 kbp, within region 2000–2,250 kbp of the Gram-positive *Streptococcus sanguinis SK36*.

The observed patterns in expressional change cannot be accounted for by expression modulation through protein binding (Figure 4), operon structure, or transcription induced supercoiling gradients, suggesting a more fundamental mechanism (Figure 5). This notion is further supported by observations of periodicities in correlation of expressional change and distance between positions of the genome in RNA-seq data (Figure 6C) from both *S. sanguinis SK36* (Data from BioProject PRJNA381491 available at GEO database; El-Rami et al., 2018), and *L. monocytogenes* (Data from BioProject PRJNA270808 available at GEO database; Tang et al., 2015). We thus speculate that the mechanism is related to the fundamental parts of the transcriptional machinery and nucleoid structure.

RNAP searches for promoters in a three-dimensional Brownian diffusion, and binds stochastically onto accessible DNA stretches until a promoter matching the associated SF is recognized (Saecker et al., 2011; Wang et al., 2013). In a condensed DNA structure, distant genomic regions may be brought within close spatial proximity, enabling the RNAP to readily diffuse between regions, thus sharing the same local pool of RNAP. Gene induction will recruit RNAP and increase the local concentration, which in turn increases the transcription initiation rate of genes in close spatial proximity. This will effectively create co-expression (Figure 5) and is visible as condensed RNAP foci during optimal growth in *E. coli*, where especially the rRNA operons are spatially clustered (Cabrera and Jin, 2003).

According to the transcriptional spilling hypothesis (Krogh et al., 2018), sharing an RNAP pool in an expanding nucleoid will cause the expression of all genes at the specific RNAP pool to drop at a similar rate. Given that the distance between the genes will increase, diluting the RNAP pool and reduce the chance of transcription initiation, and vice versa in a condensing nucleoid. This will cause spatially close genes to exhibit high correlation in pairwise expression over the cause of datasets where the nucleoid structure is modulated. If the change in distance occurs faster than the sampling rate, the pattern would be less obvious due to more noise originating from stress induction. However, if the structural changes are slowly induced within regularly structured DNA-regions, the mechanism would effectively be observable as periodic co-expressional patterns relative to distance.

If the observed sinusoidal patterns in expressional correlation are related to nucleoid structure, we hypothesize that they should match existing observations of the topology of the bacterial nucleoid. Furthermore, periodic patterns should be observable within all biological systems with nucleotide macromolecules. To test this hypothesis, the relation between DNA-DNA interaction frequencies and correlation of pairwise expression of genomic positions was investigated. The spatial proximity of DNA was determined through Chromosomal Conformation Capture (3C) data of *E. coli* during exponential growth phase, since the nucleoid is condensed during this phase, from Lioy et al. (2018). RNA-seq data was binned into 5,000 bp sized genomic bins, to match the structure of the 3C data. The correlation of pairwise expression between all unique bin pairs were computed and grouped according to the spatial proximity of the pair, respectively. Figure 6B shows the relation between spatial proximity of bins and their respective level of correlation in pairwise expression. Any relation between bins within +/−20 k bp of each other relative to genomic position was excluded to eliminate co-expression from operons and transcription-induced supercoiling gradients.

The analysis shows a genome-wide increase in correlation of pairwise expression relative to the level of measured DNA-DNA interaction. That is, the more spatially close bins are, the more they correlate in pairwise expression. This indicates that bins in spatial proximity tend to change expression levels in a coordinated manner. The observation of similar patterns in distantly related species underlines the possibility of a fundamental mechanism, related to basic systems present in most if not all living systems. It further reduces the risk of observations being an experimental bias, and to the best of our knowledge no technical bias exists in RNA-seq analysis that could cause periodic patterns in co-expression.

## Discussion and Outlook

In this study, sinusoidal patterns of correlation of pairwise expression, co-expression, with respect to genomic distance were observed in RNA-seq data. The estimated frequencies of the sinusoidal patterns were not explainable by operon structure or transcription-induced supercoiling. Furthermore, no apparent effect of most common TFs, nucleoid associated proteins, or SFs was observed.

This led to the hypothesis that the observed patterns are due to supercoiled macro structures (of more than 20 kbps, excluding immediate transcription-induced supercoiling gradients). These structures lead to spatial clusters of genes, which are distant with respect to the linear genomic sequence, but proximal in space. Such spatial clusters could potentially share RNAP pools, in which induction of genes might increase the local concentration of RNAP and thus induce the expression of other genes in spatial proximity. Underlined by the observed link between correlation of pairwise expression and DNA-DNA interaction frequencies (Figure 6). We hypothesize that spatial positioning of genes in the three-dimensional structure of the nucleoid, possible through NAPs, plays a significant role in the regulation of gene expression *via* RNAP, due to transcriptional spilling.

The size of a regular sinusoidal DNA structure with 20 kpb loops would be able to go around an averaged sized *E. coli* cell, hence we do not propose a strict sinusoidal DNA structure of the regions with patterns. But rather local flowerlike plectonemic structures, were bins at specific distances are anchored together, with long stretches of less structured DNA in between (Krogh et al., 2018). Given a length of an *E. coli* cell during exponential growth is ~2–3 μm, with a genome size of ~4.6 mbp. The length of 1 bp DNA is ~3.4 Å (340 pm), making the genome ~1.564 μm long, or 500–800 times the length of the typical *E. coli* cell. A circular stretch of 20 kbp DNA would be ~6.8 μm in circumference, with a diameter of ~2.1 μm.

In the light of recent findings showing an inconsistency between regulatory TF networks and gene co-expression, there is a gap in the understanding of how cells adapt co-expression in changing environments (Larsen et al., 2019).

A plethora of mechanisms interact and interfere with transcription of genes within complex organisms such as a bacterium. Here, DNA-structure dependent transcriptional spilling might provide an explanation for observations that do not fit in the existing paradigm of transcription regulation. Central to environmental adaption and proliferation are changes to the nucleoid structure and expressional profile of prokaryotes (Dillon and Dorman, 2010). In this perspective a fundamental regulatory mechanism utilizing structure could represent a primordial way of handling stress for DNA based organisms. Perhaps transcriptional spilling is an original simple regulatory mechanism used before more advance regulatory circuits developed, but after structural proteins such as NAPs evolved. The impact of DNA-structure on regulation of transcription is further underlined by the effect spatial distance between genes encoding TFs and the corresponding regulatory gene targets have (Pulkkinen and Metzler, 2013).

By extension, if genes are positioned in an organized manner according to expression change, it potentiates the impact location will have on insertion of synthetic DNA into the chromosome. Indeed, position-specific effects on expression of chromosomal inserted synthetic DNA have been observed (Bryant et al., 2014; Sauer et al., 2016; Scholz et al., 2019). With up to 1.000-fold differences in expression based on genomic position, it is critical to assess the insertion of synthetic DNA, and transcriptional spilling may be an important mechanism to consider. This is further supported by reduced transferability of highly expressed genes and observed proportional relation between DNA-DNA contact frequencies and transcriptional level, which suggest that highly transcribed genes have a high degree of spatial organization (Park and Zhang, 2012; Lioy et al., 2018). Highly expressed genes would exert increased constrain on the conservation of the spatial structure, since high expression yields high spilling, and thus any uncontrolled spilling could cause unwanted gene induction. This is in line with experimental data, which show a good correlation between transcription activity and stability of looped DNA domains (Dillon and Dorman, 2010).

In an evolutionary perspective, the investigation of patterns in co-expression and genomic position may unveil evolutionary forces driving optimal gene insertion and deletion. Forces dependent on the organization and conservation of distances between genes and further explain the conservation of seemingly non-sense DNA such as pseudo‐ and phantom-genes. Such genes account for ~5% of the total gene pool (225 genes; Rogozin, 2002; Keseler et al., 2013; Goodhead and Darby, 2015). These genes may act as structural elements that help the cell adjust local transcriptional spilling, both reducing unwanted spilling by acting as inactive DNA-element without recruitment of RNAP or vice versa.

It may also explain why deletion of cryptic prophage elements in *E. coli* leads to decrease in resistance to osmotic stress and antibiotics (Wang et al., 2010). If transcriptional spilling significantly influences the expressional profiles of genes it might play an important role in nucleoid structure as an evolutionary driving force. Since integration of foreign DNA, that is not optimal with regards to transcriptional spilling, might influence the ability of quick adaption to changing environments and general proliferation.

Transcriptional spilling would be another constraint that should be considered when investigating chromosomal structural evolution. In addition, this may have implications for expression of recombinant proteins *via* chromosomal insertions and may also apply to large plasmids. Especially in an evolutionary timeframe or productional framework, it could have implications in adaption and productional output. Therefore, the mechanism is highly relevant for understanding basic prokaryotic and archaic genome evolution and for optimization of cell-lines used in synthetic biology.

## Data Availability Statement

The datasets presented in this study can be found in online repositories. The names of the repository/repositories and accession number(s) can be found at: https://www.ncbi.nlm.nih.gov/geo/query/acc.cgi?acc=GSE153815.

## Author Contributions

All authors listed have made a substantial, direct and intellectual contribution to the work, and approved it for publication.

### Conflict of Interest

The authors declare that the research was conducted in the absence of any commercial or financial relationships that could be construed as a potential conflict of interest.

## Supplementary Material

SUPPLEMENTARY FIGURE S1 Correlations between genes associated to either genomic coordinate or to ordinal gene number. Plot made using data from Many Microbe Micro-arrays Database (Faith et al., 2008). Gene expression data either associated to ranked gene number based on genomic position zero, or to genomic bin based on gene coordinates. Only deviates from expression association (A-top panel) in method outlined in Figure 1.

SUPPLEMENTARY FIGURE S2 Distributions of PNmax, −log(*p*-value) of the estimated period for periods estimated within real data (Real), compared to the distribution within iterations of randomized datasets (Random), PNmax > 3, equal *p* < 0.05.

SUPPLEMENTARY FIGURE S3 Data subsets relative to expression strength and orientation, for region 800–1,050 kbp. % indicates the ranked expression strength of gene-subset.
