# Supplementary figures and images for: Elucidating the Influence of Chromosomal Architecture on Transcriptional Regulation in Prokaryotes – Observing Strong Local Effects of Nucleoid Structure on Gene Regulation
Source: Front Microbiol. 2020 Sep 1;11:2002. doi: 10.3389/fmicb.2020.02002 (PMC7491251; doi:10.3389/fmicb.2020.02002)

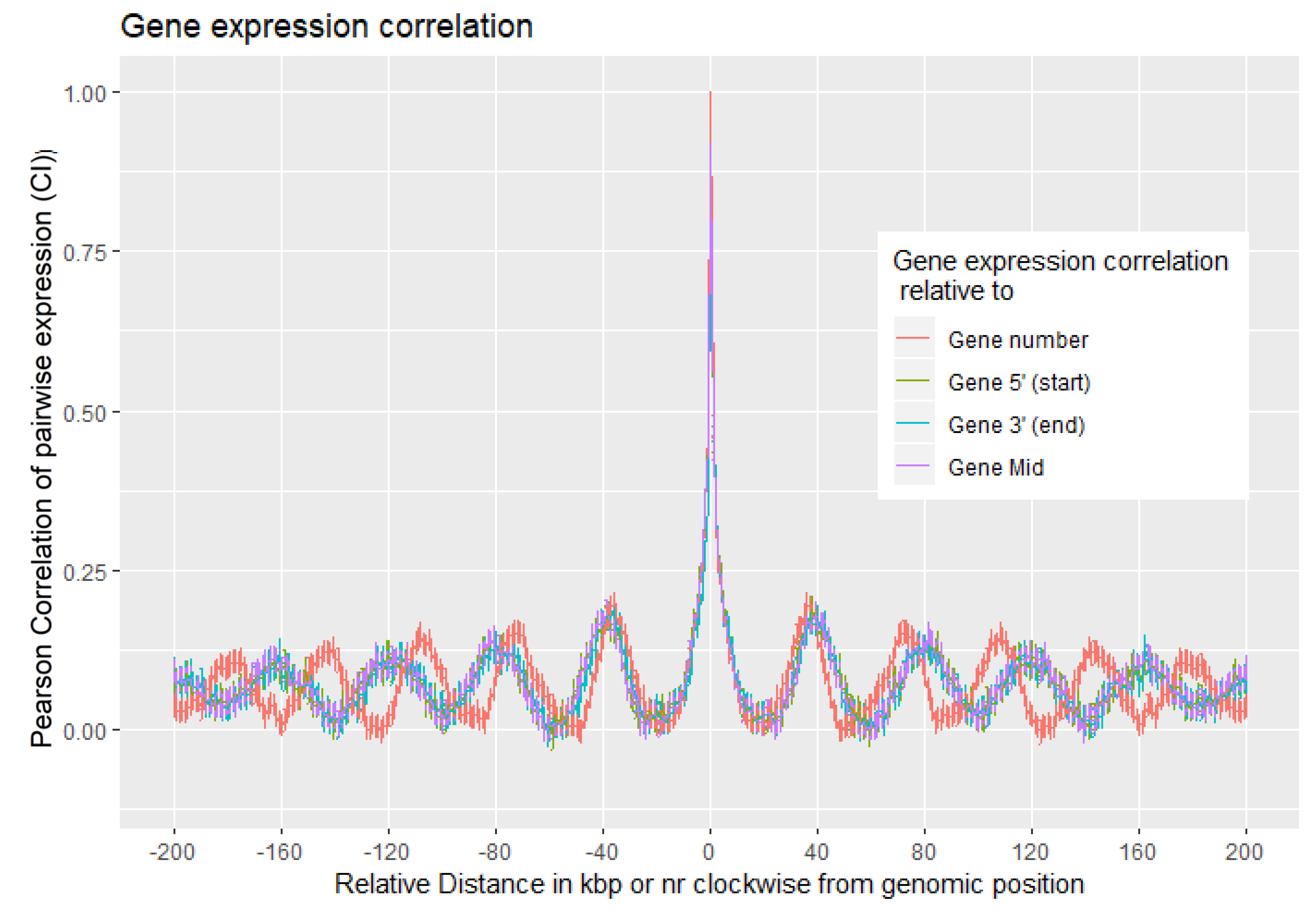

Supplement: SUPPLEMENTARY FIGURE S2 — Distributions of PNmax, −log(p-value) of the estimated period for periods estimated within real data (Real), compared to the distribution within iterations of randomized datasets (Random), PNmax > 3, equal p < 0.05. [file Image_2.TIF]

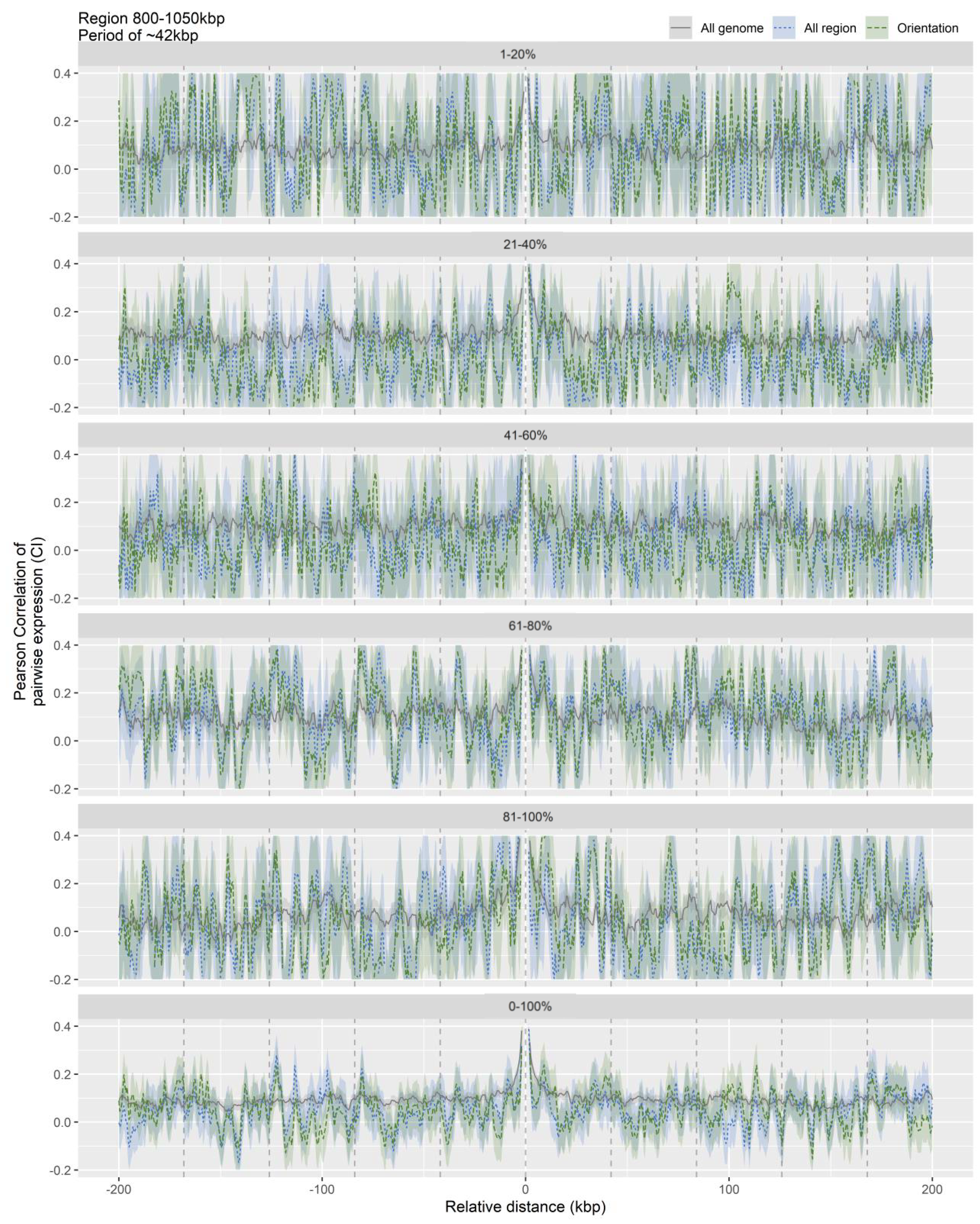

Supplement: SUPPLEMENTARY FIGURE S3 — Data subsets relative to expression strength and orientation, for region 800–1,050 kbp. % indicates the ranked expression strength of gene-subset. [file Image_3.TIF]
